# Reducing the Burden of Care: Multidisciplinary Management of Late-Manifested Crouzon Syndrome—A Case Report

**DOI:** 10.3390/children8121122

**Published:** 2021-12-03

**Authors:** Sarah Achterrath, Teresa Kruse, Julia Neuschulz, Isabelle Graf, Joachim Zöller, Bert Braumann

**Affiliations:** 1 Department of Orthodontics, Faculty of Medicine and University Hospital Cologne, University of Cologne, 50937 Cologne, North Rhine Westphalia, Germany; teresa.kruse@uk-koeln.de (T.K.); julia.neuschulz@gmx.de (J.N.); isabelle.graf@uk-koeln.de (I.G.); bert.braumann@uk-koeln.de (B.B.); 2 Center for Rare Orofacial and Craniofacial Malformations, Faculty of Medicine and University Hospital Cologne, University of Cologne, 50937 Cologne, North Rhine Westphalia, Germany; joachim.zoeller@uk-koeln.de; 3 Department of Oral and Craniomaxillofacial and Plastic Surgery, Faculty of Medicine and University Hospital Cologne, University of Cologne, 50937 Cologne, North Rhine Westphalia, Germany

**Keywords:** Crouzon syndrome, burden of care, systemic acceleratory phenomenon, quality of life, treatment planning, midface advancement, progressive postnatal pansynostosis

## Abstract

The therapy of patients with Crouzon syndrome involves a multidisciplinary team. In most cases, this therapy is extensive, time-consuming, and exhausting for the patient. This case report illustrates a temporally coordinated therapy plan that succeeds in reducing the burden of care. Showing typical extraoral characteristics of Crouzon syndrome, the patient had a frontal and left-sided crossbite, and impaction of the maxillary canines. Multidisciplinary therapy included the extraction of multiple teeth, midface distraction at Le Fort III level, and alignment of the impacted teeth. Before starting, during, and after completion of the treatment, the patient’s oral health-related quality of life was assessed using COHIP-19. The combination of different treatment steps significantly reduced the duration of therapy. The therapy improved not only the patient’s oro- and craniofacial function, but also the patient’s facial appearance in a short treatment period. The patient’s quality of life improved considerably during this time. In the treatment of severe craniofacial anomalies, the highest priority should be given to keeping the burden of care low. All measures should encourage young patients’ appropriate psychosocial development despite extensive therapies, ensuring at the same time medically satisfactory treatment results.

## 1. Introduction

Crouzon syndrome, with a prevalence of 1:60,000 [1], in most cases results from a mutation of the FGFR2 gene, which is either inherited in an autosomal dominant manner or arises as a de novo mutation, as in 30–60% of cases [2,3,4,5]. Premature cranial suture closure results in growth inhibition perpendicular to the affected suture [6]. Craniosynostosis of the coronal and sagittal sutures, premature fusion of circummaxillary sutures, and inhibited growth of the skull base lead to complex facial deformities in patients with Crouzon syndrome [7]. Compensatory growth parallel to the fused sutures is consequently increased [6]. Among all craniosynostoses, Crouzon syndrome is the most common [8].

Clinical extraoral characteristics of Crouzon syndrome include brachycephaly, midface hypoplasia, maxillary retrognathia and micrognathia, a prominent nose, exophthalmos, strabismus, and hypertelorism [2]. Permanently increased intracranial pressure due to prematurely occluded sutures can lead to compression of the optic nerve with the risk of a subsequent atrophy. Hearing loss in more than half of the patients and mental retardation were described [2,9,10]. Intraoral characteristics are a possible cleft palate and a narrow maxilla. This can lead to extreme crowding and disturbed eruption of the upper canines [9,11].

The phenotypic appearance of patients with Crouzon syndrome varies and is influenced, among other things, by the timing of suture synostosis [12]. Recent reports described a subtype of craniosynostosis characterized by progressive postnatal pansynostosis [13,14]. In fact, premature fusion should be regarded as a dynamic process [4]: postnatal growth and head shape may initially be regular. A lack of increase in head circumference in newborns, however, should be considered a serious sign, as a dramatical increase in intracranial pressure can occur with sutural fusion. Regular measuring of the head circumference allows for, among other things, an early indication of the presence of craniosynostosis [14]. Patients with late-manifested craniosynostosis, mainly diagnosed clinically in the course of growth, may form a distinctive subgroup of Crouzon syndrome [15], but could not be assigned to a specific mutation by human genetics so far [12].

The multidisciplinary therapy of patients with Crouzon syndrome poses great challenges to oral and maxillofacial surgeons, pediatricians, ear, nose, and throat specialists, ophthalmologists, human geneticists, clinical psychologists, speech therapists, and orthodontists. Although surgical distractions or one-step osteotomies are promising and provide clear benefits for the patient, they cannot normalize growth in the pathologically occluded sutures. However, in a time-limited fashion, such surgery stimulates bone metabolism both locally and systemically [16,17]. With good timing, this phenomenon can be used orthodontically to shorten the duration of therapy, as accelerated tooth movement results [18].

The WHO has set the global goal of reducing the burden of care for patients with craniofacial anomalies [19]. The term “burden of care” describes a burden arising in the context of therapy for the patients themselves, but also for their family environment.

The following case report illustrates a time-efficient, burden-of-care-reducing treatment plan for a patient with Crouzon syndrome and impacted canines.

## 2. Case Report

**Figure 1:**
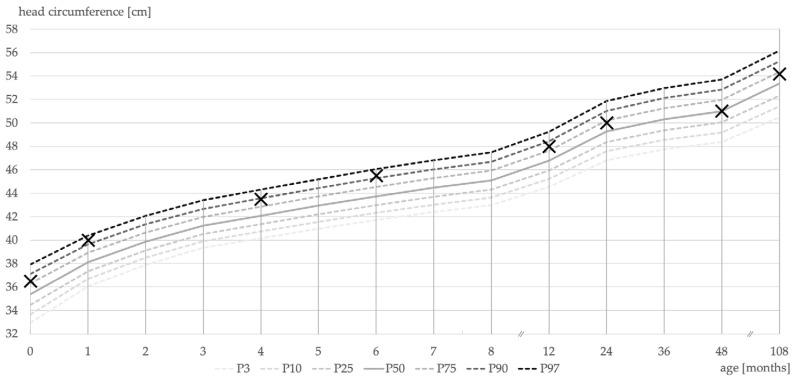
Percentile curves (P3–P97) of head circumference as a measure of dispersion. Crosses mark the patient’s measured head circumference during routine examinations. Patient’s head circumference was above the 50th percentile during his first 9 years of life.

Postnatally, the patient was presented to the clinic for General Pediatrics at the University Hospital of Cologne. The parents described the patient’s existing respiratory problem. Apart from nasal cavities narrowed on both sides, neither magnetic resonance imaging (MRI) nor computed tomography of the skull revealed any pathological findings at this time. Sonographically, the patient’s cranial sutures appeared open. The patient’s head circumference was continuously monitored and was within normal limits (Figure 1).

Due to the persistence of respiratory problems, limited masticatory function, snoring issues, and a differing external appearance, the patient was presented to the Center for Rare Orofacial and Craniofacial Malformations at the University Hospital of Cologne around the age of seven. During a detailed clinical examination by oral and maxillofacial surgeons, pediatricians, and ophthalmologists, the diagnosis of Crouzon syndrome was confirmed by human geneticists. A de novo mutation in exon 7 of the FGFR2 gene (p.Tyr281Cys) in the heterozygous state was revealed.

**Figure 2:**
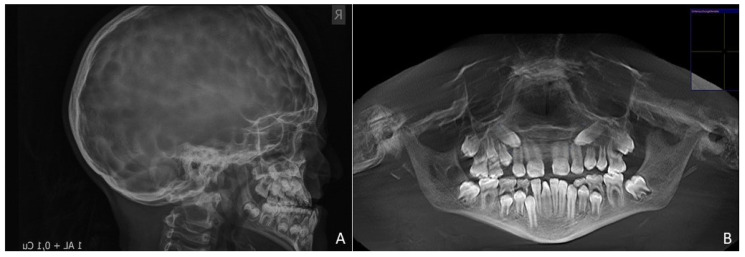
Pretreatment radiographs. (**A**). Lateral cephalogram showing extreme mesial–basal jaw relationship. Prominence of convolutional markings throughout the neurocranium indicates chronic increase in intracranial pressure. (**B**). Panoramic view of a digital volume tomogram showing impaction of maxillary canines and short skeletal base of micrognathic maxilla.

A “copper beaten skull” was evident upon radiographic examination (Figure 2A). An MRI of the skull showed overcrowding due to premature suture synostosis, indicating a mismatch between the volume of the cerebellum and that of the posterior fossa of the skull. Well-developed lateral cerebral ventricles showed that no threatening intracranial pressure was present.

**Figure 3:**
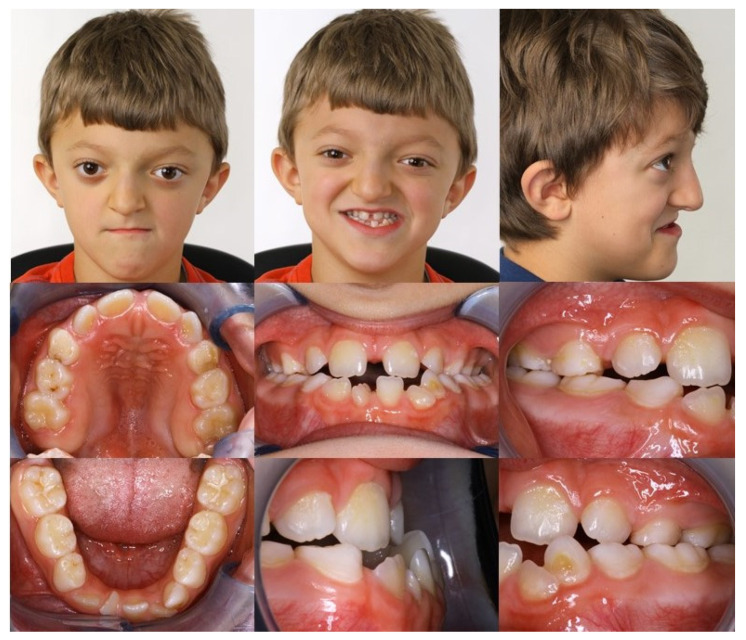
Intraoral and extraoral findings of the patient at initial examination. Extraoral: brachycephalus, exophthalmos, hypertelorism, and underdevelopment of the midface with absent zygomatic prominence. Intraoral: high palatal vault, pronounced crowding in the upper and lower jaw, anterior and posterior crossbite, and Class III malocclusion.

Extraorally, the typical Crouzon syndrome features of brachycephalus, exophthalmos, and hypertelorism were notable. In profile view, the underdevelopment of the midface with absent zygomatic prominence was evident (Figure 3). Intraorally, an early mixed dentition with pronounced crowding and complete loss of space for the upper permanent canines was observed. The maxilla appeared narrow with a high palatal vault without a cleft palate (Figure 3). The extreme mesial–basal jaw relationship came with a WITS appraisal of −18.9 mm, an extremely retrognathic maxilla, and a minor retrognathic mandible (Figure 2A). The need for treatment was manifested intraorally by a more than a full-step class III malocclusion. The patient showed an anterior and left-sided crossbite, and a tendency towards an open bite (Figure 3). Radiographically, impaction of the maxillary canines was evident (Figure 2B).

**Figure 4:**
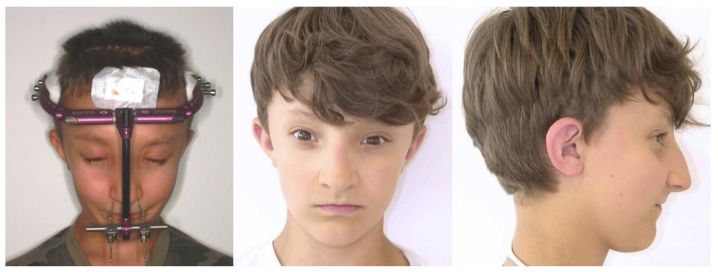
Midface distraction using RED distractors (left) and extraoral result immediately after distraction. To correct the temporary open bite, the distraction vector was adjusted caudally. As residual growth was expected, overcorrection of the midface advancement was performed.

At the start of orthodontic therapy, the patient was eight years old. A functional appliance was used for pretreatment and orofacial stimulation. The extraction of deciduous teeth and upper first premolars had been performed before a multibracket appliance was inserted. Eight months later, the lower first premolars were extracted, and the maxillary canines were surgically exposed in the same procedure. The orthodontic extrusion of the canines was intentionally combined with the successive midface distraction. A modified Le Fort III osteotomy was performed with subsequent midface distraction by a rigid external distraction device (RED distractor) fixed to the cranial vault: after intraoperative mobilization of the facial skeleton, tensile wires were fixed to the apertura piriformis penetrating through the skin laterally to the nose (Figure 4).

**Figure 5:**
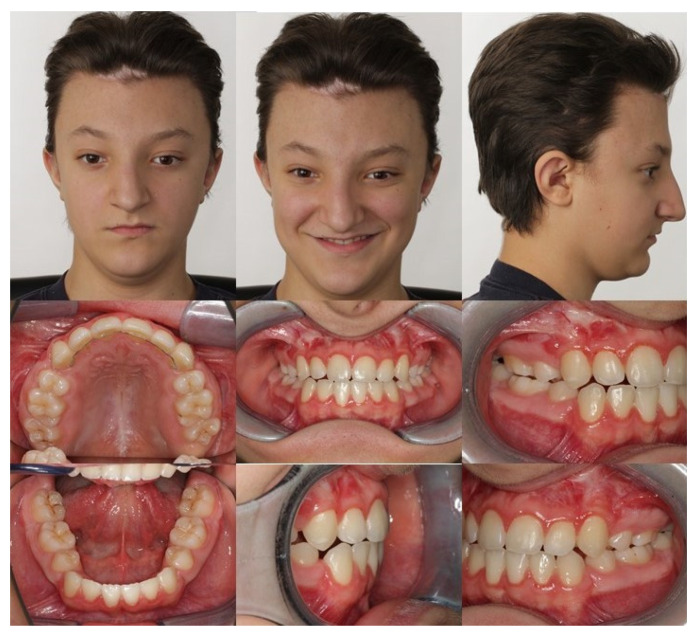
Stable treatment result at the end of the orthodontic retention period at the age of 15. A slight relapse of the skeletal (and thus also dental) relationship due to permanent growth inhibition in the midface region cannot be ruled out until definitive growth completion.

**Figure 6:**
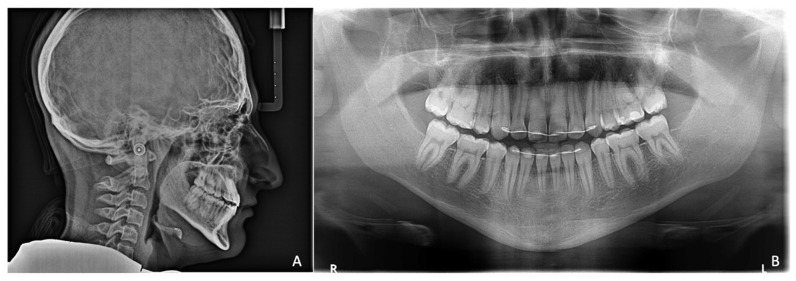
Posttreatment radiographs at the age of 15. (**A**). Lateral cephalogram at the end of the orthodontic retention period showing a slight relapse of the skeletal relationship. (**B**). Orthopantomogram showing the stable result with aligned canines.

Distraction started seven days postoperatively by using a RED distractor and activating it twice daily for 14 days. A distraction rate of one millimeter per day was thus ensured [20]. In the subsequent ten-week retention phase, the traction wires remained fixed in the rigid external device, and retention by means of a face mask was therefore unnecessary. The multibracket appliance was in situ for a total of two years, followed by an orthodontic retention period of 28 months. At the end of the orthodontic therapy, the patient showed a well-balanced facial profile. Upper and lower dental arches were harmonized and stabilized in Class I occlusion (Figure 5). The slight but stable overjet fitted the minor skeletal Class III pattern, which was not fully corrected (WITS −5.3 mm, Figure 6). The patient was very adherent and attended a total of 33 orthodontic appointments during active treatment.

### Assessment of Patient’s Quality of Life

In addition to physical limitations, patients with Crouzon syndrome face extensive psychological and social challenges. Anomalies in the outer appearance can lead to social anxiety and lack of self-confidence in affected individuals [21]. Among patients with syndromal craniosynostosis, those with Crouzon syndrome have severely reduced quality of life. Due to lower mental limitations compared to patients with Apert syndrome, they participate more extensively in mainstream society. Emotional pressure is perceived more intensely [22].

The extensive medical treatment necessary for patients with Crouzon syndrome affects their quality of life. Initially, the young patients and their parents may have difficulties in understanding the complexity of treatment. At the same time, it requires the parents to make serious decisions for the future of the child’s health. The goal of treatment should therefore always focus on a verifiable improvement in the patient’s quality of life pursuing at the same time an individual functional and aesthetic optimum [23]. Weighing these two aspects during therapy helps to continuously adapt the therapy concept to the individual needs of the patient.

A reliable and valid method for measuring oral health-related quality of life is the COHIP-19 (19-item version of the Child Oral Health Impact Profile) questionnaire. This questionnaire allows for conclusions to be drawn about oral health, functional and social–emotional well-being, school environment, and the patient’s self-image [24]. By answering 19 questions, a score between 0 (worst oral health-related quality of life) and 76 (best oral health-related quality of life) is determined. Before any treatment, the patient’s COHIP-19 score was 55. Compared with children and adolescents of similar age undergoing orthodontic treatment (COHIP-19, 63.2 points), his quality of life appeared reduced [25].

In order to prevent a further reduction in the patient’s quality of life, the timing of the midface distraction had to be critically considered. From a surgical point of view, the ideal age is between the 7th and 14th year of life [4,26]. Practitioners and patients’ parents hope that surgery before puberty would increase the child’s self-confidence and facilitate social integration. The possibility of a postoperative “fresh start” in the patient’s social environment also speaks in favor of prepubertal surgery [27].

The average duration of distraction osteogenesis is three months including the retention phase [28]. In this case, the timing of the midface distraction was coordinated with the summer vacation of the then 12-year-old patient. With psychotherapeutic support, the patient agreed to return to school before completion of the retention phase.

Oral health-related quality of life assessed during treatment did not decrease. Two years after removal of the multibracket appliance and with completion of the retention phase, the patient’s quality of life was re-evaluated and increased to a value of 74 out of a possible 76 points (i.e., an increase of 25%).

## 3. Discussion

The diagnosis of Crouzon syndrome and treatment of these patients are left to a few specialized centers and often challenge even experienced practitioners. Without the typical ossification of the sutures in the first year of life, the diagnosis of Crouzon syndrome may be delayed, as in this case. Contrary to patients with prematurely occluded sutures at birth, this rare subtype of progressive postnatal pansynostosis does not require such acute treatment postnatally as long as the head circumference and/or the intracranial pressure are continuously monitored. Monitoring intracranial pressure in a noninvasive, radiation-hygienic and clinically practicable way is still a major challenge for specialists. Measuring head circumference is a clinically practical method [14]. If the head circumference decreases, a CT scan is indicated [13,14]. In our patient, there was no reduction in head circumference in the first years of life (Figure 1). No fronto-orbital advancement was indicated in this patient. Midface hypoplasia, which accentuated during growth, indicated a Le Fort III osteotomy with distraction—a therapy that is superior to one-step methods in terms of improving the patient’s external appearance [28]. The application of tensile forces to the apertura piriformis provides an individually modifiable distraction vector near the center of rotation or the center of resistance [20,29]. During distraction osteogenesis, a modification of the vector in a caudal direction counteracted the counterclockwise rotation of the maxilla.

In the presented case, repeated surgical procedures in a short period of time were weighed against a reduction of the total therapy duration. Surgical exposure of the impacted canines two months prior to distraction appeared to be most reasonable for burden of care reduction. Two months after removal of the RED distractor, orthodontically assisted eruption of the canines was complete. A clinically correct position of the canines was reached in a total time of seven months, which was less than the average [30]. The simultaneous distraction opened the space for aligning the canines and changed the metabolic activity of the bone. This phenomenon occurs both locally (regional acceleratory phenomenon) and systemically (systemic acceleratory phenomenon) [16,17]. The reduced bone density postoperatively increases remodeling [31] and accelerates tooth movement during and up to 3–4 months postoperatively [18]. This could be used to reduce the active treatment time. Premature fusion also of the circummaxillary sutures in these patients is frequent. Orthopedic treatment on the maxilla (e.g., palatal expansion and/or protraction) without surgical assistance should be planned with caution. A computed tomography is recommended to verify the patency of sutures to avoid unnecessarily frustrating and dangerous trials, that increase the burden of care [7].

A well-structured therapy concept with special attention to the burden of care can simultaneously reduce the patient’s physical and psychological suffering. In addition to medical indications, patients’ developmental resilience and their school obligations are taken into account in the structuring of the therapy.

Even with a stable treatment outcome as in this patient, relapse cannot be prevented in every case despite intensive retention measures. In patients with Crouzon syndrome, another surgical intervention is sometimes necessary at about 18 years of age due to permanent growth inhibition in the midface sutures [32].

## 4. Conclusions

A coordinated therapy plan for patients with Crouzon syndrome that sensibly integrates the orthodontic treatment steps can significantly shorten their therapy. By taking into account age-related and individual life circumstances, the therapy concept should meet the patient’s multifaceted needs. In the presented case, coordinated planning and time-efficient achievement of the therapy goals resulted in an increased quality of life and a reduced burden of care. Due to the late need for intervention, the therapy could be kept very compact. In order to further improve therapy structuring for patients with Crouzon syndrome, detailed research on subtypes of this syndrome, possibly also at the human genetic level, would be beneficial.

In any multidisciplinary treatment of craniofacial anomalies, patient-related factors should be considered in addition to the coordination of the purely medical aspects of treatment.

## Data Availability

The data presented in this study are available on request from the corresponding author. The data are not publicly due to protection of privacy.
